# Insights in the Effect of Fluctuating Female Hormones on Injury Risk—Challenge and Chance

**DOI:** 10.3389/fphys.2022.827726

**Published:** 2022-02-17

**Authors:** Kirsten Legerlotz, Tina Nobis

**Affiliations:** ^1^Movement Biomechanics, Institute of Sport Sciences, Humboldt-Universität zu Berlin, Berlin, Germany; ^2^Berlin Institute for Integration and Migration Research, Humboldt-Universität zu Berlin, Berlin, Germany

**Keywords:** estrogen, menstrual cycle, gender bias, injuries, women, sport, exercise, ligament

## Abstract

It is time to take on the challenge of investigating the complex effect of fluctuating female hormones on injury risk as this offers a chance to improve female athletes’ health and performance. During the recent decade, the body of knowledge on female hormones and injury risk has largely been increased. New insights have been offered regarding the association of certain phases of the menstrual cycle and injury prevalence as well as regarding relationships between hormone levels and musculoskeletal changes such as, for example, ligamentous stiffness and knee laxity. However, current research often follows the theme of a causal relationship between estrogen levels and musculoskeletal function or injury and thus—one might argue—further enhances a rather simplistic approach, instead of uncovering complex relationships which could help in establishing more nuanced ways of preventing female injuries. To uncover real effects and to truly understand the physiological responses, we suggest to reflect on potential bias regarding research questions and current approaches. It may enhance future studies to apply a more nuanced approach to causation, to include multidimensional perspectives and to implement an interdisciplinary methodology.

## Introduction

It is well accepted that females are currently less represented as research subjects in exercise physiology studies (Costello et al., 2014). Most probably, reasons for this imbalance are, that fluctuating female hormones are thought to generate less-controlled experimental conditions and to increase variation thereby reducing statistical power and making studies more complex and expensive (Uhl et al., 2007). As a result, specific knowledge on women’s response to exercise is limited, hindering our understanding of female exercise physiology. This imbalance in research does not hold true for all fields of exercise physiology studies. Changes in bone health as a result of hormonal and metabolic changes due to insufficient energy intake and/or excessive exercise energy expenditure, known as the female athlete triad, have predominantly been investigated in women. Today, it is accepted that detrimental effects of energy deficiency, respectively, the relative energy deficiency syndrome are not limited to exercising girls and women and but are also occurring in men (de Souza et al., 2021). However, the overall imbalance in research cannot be neglected, which also raises ethically relevant questions about whether women receive lesser chances of performance progression and about whether women are subjected to a higher risk of injury compared to men due to a limitation of specific knowledge. Indeed, it has been shown for injuries sustained in car accidents, that protective concepts, which have been based on the biomechanical behavior of male bodies only, critically impact the safety of women in everyday life (Linder and Svedberg, 2019).

Consequently, it can be argued that the specific relevance of studies about female exercise physiology which consider hormonal fluctuations lies within their potential of providing applied knowledge on how to prevent injuries and improve performance in exercising women. In light of this starting point, this article sheds light on the progress made in female exercise physiology, namely on how research and researchers deal with the menstrual cycle and injury risk and it furthermore identifies and discusses current and future challenges in this field of research. We will particularly focus on ACL injuries, using this type of injury as an example to demonstrate the specific achievements and challenges associated with menstrual cycle-related research in exercising women. In a first step, we will review current studies on this topic by portraying their research questions, results, and explanatory approaches. As a result, we will show that the body of knowledge about the menstrual cycle and injury risk has risen significantly. However, some challenges, which we will critically reflect and discuss in the second part of this paper, remain to be acknowledged and addressed. Secondly, as a result, we will unfold the thesis that we—as researchers—might not always be as objective as we often assume to be and that we furthermore tend to simplify the complex relationship between the menstrual cycle and injury risk. Having identified and discussed these shortcomings, we will consequently outline perspectives and strategies for future research in the conclusion of this article.

## State of Research

### Female Hormones and Injury Risk

Exercise-associated injuries are accompanied by a variety of side effects. Besides generating costs for the healthcare system, they may affect the injured athletes’ quality of life or their athletic careers (Palmer et al., 2021). This insight often leads sport scientists to the value-driven desire of developing strategies that reduce the prevalence of those injuries. In this context, one approach is to identify risk factors for specific injuries, which then could be addressed with corresponding interventions.

Some of the injuries frequently occurring during exercise activity are more prevalent in women than in men (Waldén et al., 2011; Lovalekar et al., 2020), leading some to suggest that female sex itself is a risk factor. As physiological differences between men and women are to a great extent a result of a differing hormonal profile, it suggests itself to take a closer look at the effect of female sex hormones. It has long been known and frequently been reported for female athletes, that permanently low levels of female sex hormones, accompanied by menstrual cycle perturbations such as amenorrhea, are detrimental for bone health and increase the risk for stress fractures (Olson, 1989). The incidence of menstrual cycle disturbances is thereby substantially higher in athletes than in the general population, particularly in aesthetic and weight-bearing sports (Redman and Loucks, 2005).

In eumenorrheic exercising women, the blood plasma concentration of female sex hormones naturally fluctuates during the menstrual cycle (Pirke et al., 1990). Thus, one line of research about the effect of female sex hormones focuses on the correlation between the menstrual cycle phase and injury risk, trying to establish if hormone level fluctuations lead to corresponding fluctuations in injury risk (Martin et al., 2021). Indeed, if we look at ruptures of the anterior cruciate ligament (ACL), not only will we observe, that ACL ruptures are much more prevalent in women than in men (Myklebust et al., 1998; Waldén et al., 2011), but also that they are associated with certain phases of the menstrual cycle (Myklebust et al., 1998; Wojtys et al., 2002; Ruedl et al., 2009; Lefevre et al., 2013).

### Menstrual Cycle and ACL Ruptures

Due to the striking difference in sex-related injury prevalence, with a 2–3-fold elevated relative injury risk in women compared to men (Waldén et al., 2011), the ACL rupture may be the best investigated of all sex associated sport injuries. In addition, it can be argued that the high medical burden of ACL injuries makes it all the more urgent to develop strategies, respectively, recommendations that can effectively reduce injury prevalence specifically in women. Thus, many studies have investigated if the occurrence of ACL ruptures is related to the hormonal profile and hence differs between the follicular, the ovulatory and the luteal phase of the menstrual cycle. Several reviews and meta-analyses, surveying five (Herzberg et al., 2017), seven (Hewett et al., 2007), and nine (Somerson et al., 2019) studies, have since concluded that there is a decreased relative risk of an ACL tear in the luteal phase, respectively, that female athletes are more predisposed to ACL ruptures during the preovulatory phase of the menstrual cycle. The finding, that there is an association of ACL ruptures with the preovulatory phase, as detected in a study describing the prevalence of ACL ruptures along the menstrual cycle during skiing, may only allow for rather general recommendations such as “Female skiers should take special care during this period” (Lefevre et al., 2013). One might ask if this actually is a realistic point of action, suited to reduce injuries. To allow for more precise recommendations, which are derived from addressing the reasons (and not the outcomes) for variations in injury prevalence, we need to uncover the mechanisms behind the association of menstrual cycle phase and injury prevalence.

### Hormonal Fluctuations, Ligamentous Properties, and Knee Laxity

**Figure 1:**
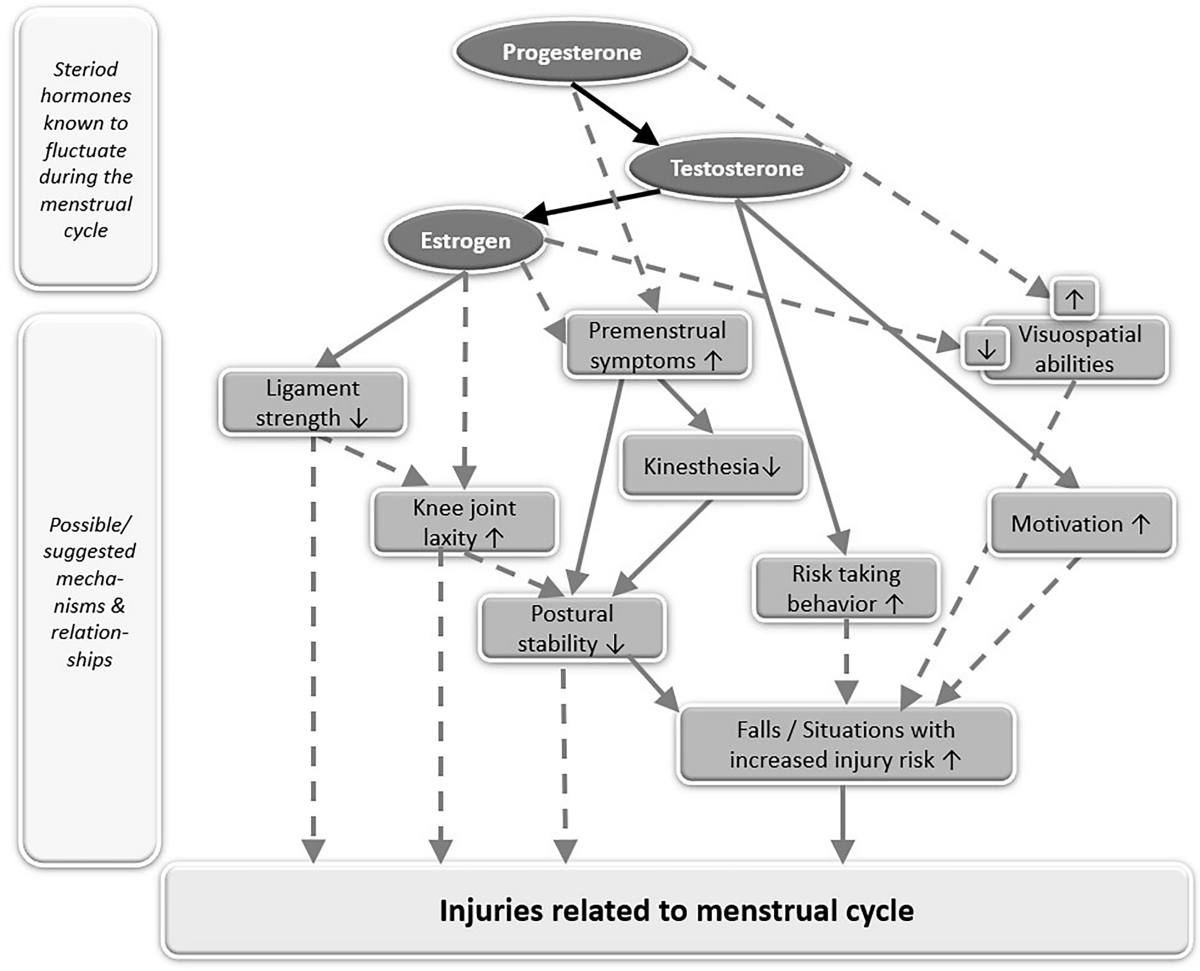
Simplified model of known (solid grey arrows) or suggested (broken grey arrows) relationships between steroid hormones, fluctuating during the menstrual cycle, and their effects on the musculoskeletal and neurophysiological system, assumed to be related to injury risk. Upwards pointing arrows within the boxes symbolize an increase while downwards pointing arrows within the boxes symbolize a decrease. Black arrows connecting progesterone, testosterone, and estrogen symbolize the biosynthetic pathway of steroid hormones.

The most popular explanation for the statistical correlation between preovulatory phase and injury peak is to directly link high estrogen levels to a mechanical weakness of ligaments, which would then—in turn—increase the likelihood for ACL injuries (Figure 1). It has been assumed, that female hormones, and in particular estrogen, make female ligaments susceptible for injury by directly affecting ligamentous metabolism (Wojtys et al., 2002; Chidi-Ogbolu and Baar, 2018). It has also been suggested, that the ligamentous response to estrogen may be related to an evolutionary adaptation as decreased connective tissue stiffness and laxer joints would facilitate healthy childbirth (Chidi-Ogbolu and Baar, 2018). However, when taking a closer look at this assumed causal relationship between high estrogen levels and weak, more compliant or less stress-resistant ligaments, recent study results raise the question whether this causal explanation is too naïve. One might argue, that when pregnancy actually occurs and estrogen levels are much higher, an evolutionary advantageous decrease in connective tissue stiffness must be much more pronounced. However, there is no evidence to support this. In contrast, stiffness of the ligamentum patellae does not change during pregnancy and it neither differs between pregnant women and non-pregnant controls (Bey et al., 2019). Similarly, no difference in biomechanical properties of the ligamentum patellae, fibril characteristics, or collagen cross-linking was observed between the different phases of the menstrual cycle or between women using oral contraceptives and those who did not (Hansen et al., 2013). While these results do not necessarily or finally falsify the assumption about the causal relationship between estrogen levels and ligamentous stiffness, they at least raise serious concerns about its effect size.

The assumption, that female hormones make ligaments susceptible for injury, leads us to a second causal relationship, which has often been proposed and which links high estrogen levels to increased knee laxity, which would then—in turn—increase the likelihood for ACL injuries (Figure 1). The concept, that ligamentous stiffness of knee joint structures decreases with rising estrogen levels, is related to the observation of increased knee joint laxity when estrogen levels are high during the menstrual cycle (Heitz et al., 1999) and during pregnancy (Schauberger et al., 1996). As knee joint laxity is thought to increase injury risk, several studies have been conducted to investigate the relationship of knee laxity, injury risk, and the menstrual cycle. Two meta-analyses reviewing 9 (Zazulak et al., 2006) and 19 studies (Somerson et al., 2019) have since concluded, that knee laxity indeed varies within the menstrual cycle, with the highest laxity occurring during ovulation, followed by the luteal phase and the lowest laxity occurring in the follicular phase. However, both meta-analyses also concluded, that periods of increased laxity were not associated with an increased prevalence of ACL ruptures (Zazulak et al., 2006; Somerson et al., 2019), suggesting that there is no direct link between hormonal effects on ACL injuries and hormonal effects on knee laxity. It may further be questioned if transient non-pathological changes in healthy women’s knee laxity have a pronounced effect on injury risk, as a 3-year prospective cohort study with young female athletes could not detect a difference in knee laxity between those who subsequently teared their ACL and those who did not (Nakase et al., 2020). It also has to be considered, that tibial translation, commonly described as knee joint laxity, is no direct measure of the ACLs mechanical properties. While tibial translation has been found to inversely correlate with ultimate failure strength of ACL grafts in animal studies (Beynnon et al., 1994), other variables than ligamentous stiffness may also contribute to knee stability. Knee laxity might change without a change in ligamentous stiffness as an animal study with pregnant rabbits suggests (Hart et al., 2000). There, pregnancy was associated with increased laxity while it had no effect on the structural (stiffness and failure load), material (stress at failure and Young’s modulus), or viscoelastic (cyclic and static relaxation) properties of the medial collateral ligament (Hart et al., 2000).

### Menstrual Cycle and Postural Stability

While hormonal-induced increases in knee laxity may not directly be related to ligamentous stiffness and may not directly affect injury risk, they could have an indirect effect by impairing postural control (Figure 1). However, there is no consensus as to if and when postural control is impaired during the menstrual cycle, as studies found both improved (Emami et al., 2019) and impaired (Lee et al., 2017) postural control at ovulation or no change in postural control during the menstrual cycle at all (Legerlotz et al., 2018). One explanation for those varying results may be, that pronounced hormonally induced impairments in postural control only occur in a specific subpopulation of women: those who suffer from premenstrual symptoms (Fridén et al., 2003, 2005). These women showed increased postural sway in the luteal phase, when symptoms like discomfort and pain were particularly prevalent, while sway values stayed stable throughout the menstrual cycle in women without symptoms (Fridén et al., 2005). That postural stability in women with premenstrual symptoms is generally reduced compared to women without symptoms may be related to changes in kinesthesia, as women with premenstrual symptoms also displayed a greater threshold for detection of passive movement in the knee (Fridén et al., 2003).

### Menstrual Cycle and Cognitive Functioning

Hormonal changes during the menstrual cycle can not only affect physical and functional variables, such as knee laxity, postural control or kinesthesia, which are related to movement control. They can also affect the main instance of movement control itself, our brain. Sex hormones may have a far-reaching impact on brain function, with both behavioral consequences as well as changes in neuropsychological processing (Hornung et al., 2020). It has been suggested that healthy women show small fluctuations in cognitive performance across the menstrual cycle, with low-performance scores in the luteal phase for visuospatial and motor skills, attention and concentration, verbal memory, visual memory, working memory, and reaction time (Souza et al., 2012). However, associations between prefrontal cognitive functioning and hormone levels across the female menstrual cycle detected in one cycle can not necessarily be replicated when analyzing a second cycle, suggesting the occurrence of false-positive findings attributable to random variation particularly in small samples (Leeners et al., 2017). Sex differences in visuospatial abilities have long been reported, with men usually performing better in the Mental Rotations Test, and women with low estrogen levels performing as strongly as males (Peragine et al., 2020). In contrast, a recent study with a large sample size of 528 women found no effect of estrogen on mental rotation performance, while high levels of progesterone, characteristic of the luteal phase, were instead associated with small performance increases (Shirazi et al., 2021). In general, the observed effects are small and not consistent and should thus be treated with caution.

### Menstrual Cycle and Behavior

Hormone-associated changes in brain function may also change women’s behavior during the menstrual cycle, which could as well have an impact on injury prevalence. The motivation to train and the motivation to compete have both been shown to be elevated around ovulation (Cook et al., 2018). It may be speculated, that increased motivation potentially changes the characteristics of movement, such as movement intensity, thereby affecting the risk of injury (Figure 1). In addition, behavioral experiments have indicated that risk-taking behavior also changes along the menstrual cycle, with women being willing to take higher risks around ovulation (Cook and Crewther, 2019). It seems likely, that increased risk-taking behavior may positively affect performance and competition outcome while at the same time increasing the risk of injury.

## Discussion

We acknowledge the great efforts that have been made to uncover relationships between female hormones, musculoskeletal properties, neurophysiological changes and injury risk, the fact that the body of knowledge has largely been increased, and new insights have been offered. However, when taking a closer look at this particular field of research several questions remain to be unanswered, several contradictions yet to be solved, several correlations yet to be explained. Research often follows the theme of a causal relationship between estrogen levels and musculoskeletal function or injury and thus—one might argue—further enhances a rather simplistic approach, instead of uncovering complex relationships which could help in establishing more nuanced ways of preventing female injuries. With this specific evaluation of the state of research in mind, we will discuss possible reasons for these shortcomings and identify three themes, respectively, approaches that require some further elaboration:

1. **Deficit-oriented approach:** The effects of female sex hormones appear to be seen and valued in light of weakening the women’s body, with females being—be it consciously or unconsciously—viewed as less functioning, weaker or less well-adapted men. In the context of the menstrual cycle research often appears to start with the underlying—yet often implicit—assumption that female hormones, the menstrual cycle or the menstrual bleeding are seen as something avoidable, as something “special” or as something undesirable. As a consequence, it is assumed that female hormones or the menstrual cycle are responsible for higher rates of specific injuries or reduced performance. This deficit- and problem-oriented approach is mirrored in the underlying assumptions that often initiate research and is reflected by the mechanistic approach that has been applied to link female sex hormones such as estrogen to mechanical or functional weakness (Chidi-Ogbolu and Baar, 2018), interpreting physiological changes observed during the menstrual cycle as negative, and associating those changes with an increased risk of injury. Research that focuses on possible, positively associated or protective effects of female hormones appears to be rare.
2. **Oversimplification:** Although the literature strongly suggests that there is an association of ACL ruptures with the preovulatory phase, this by itself does not explain, why the injury prevalence varies between phases, but it rather begs the question “why.” It is crucial to not fall into the trap of mistaking a correlation for a causation, of overseeing the possibility that statistical effects can also be caused by a third variable, only hold true for some subgroups, or that further variables could be way more relevant than the one regarded in one’s own statistical model. Thus, to uncover the mechanism behind peaks in injury incidence during the menstrual cycle it is important to understand, that it is not exclusively the estrogen level that varies with different phases of the menstrual cycle. Other variables, such as, for example, the testosterone level similarly vary with different phases of the menstrual cycle (Cook et al., 2018; Cook and Crewther, 2019). Further physiological, biomechanical, functional, but also psychological and behavioral variables need to be considered in interdisciplinary studies to uncover the complex relationship of injury risk in female exercise. In addition, it may be too simplistic to view women as one homogenous group, particularly in relation to hormonal variation. Hormone levels can vary greatly between female elite and non-elite athletes (Cook et al., 2018), and different subpopulations of women, for example, those suffering from premenstrual symptoms, may adapt or react differently (Fridén et al., 2003, 2005).
3. **Researcher bias:** We argue that current research approaches aiming to investigate the relationship of female hormones and musculoskeletal adaptation or injury risk indicate that we—as researchers—might not be as objective as we often claim to be. Besides from being researchers, academics or sport scientists we always also are and always will be members of societies in which we have been socialized and through which we have incorporated a specific set of norms and values that also imply gender-related issues. Methodological choices and criteria for evidential support may not involve value commitments—even though this too is a rather controversially discussed topic within science theory (Lacey, 2018). However, it has long been discussed and approved that whichever research question we claim is to be more relevant than another and whichever reasons we find for this relevance is a value-based decision that is likely to be correlated to our position in society (Homans, 1976; McMullin, 1982; Lacey, 2018). Furthermore, and knowing that our views on society and on specific topics as well as our experiences are also—yet not exclusively—shaped by the gender we identify with or that is ascribed to us, leads us to ask whether the deficit- and problem-oriented approach to female physiology might also be a result of a male-dominated research field.

## Conclusion: Accepting the challenge

Shifting the focus from the status quo and its evaluation to perspectives for research, one could argue that studying and understanding the complex effects of the menstrual cycle on injury and performance remains a task yet to be fulfilled, yet also a task that can build on the achievements of prior research in this field. Following our line of argumentation leads us to the following, more nuanced recommendations:

- Self-reflection on potential bias and a more nuanced approach to causation can be seen as crucial points of action for future research. Raising research questions that help to uncover the supposedly multiple factors that contribute to injury risks and that lead researchers to also include positively associated or protective effects of female hormones in their studies, implementing approaches that acknowledge the great differences within the group of women and a more careful interpretation of results can be regarded as relevant tasks for future research.
- Multidimensional and interdisciplinary research is strongly needed. To uncover real causal effects and to truly understand the direction of hormonal effects as well as the meaning and the significance of these effects, requires to put them into perspective. Interdisciplinary research that simultaneously—and not separately—considers multiple factors can help to achieve this. For example, postural stability variations during the menstrual cycle can be affected by a variety of variables such as neurophysiological or visuospatial abilities, psychological state (anxiety) and tissue mechanical properties, which all need to be considered within the same study.
- Accounting on sex and gender, not only regarding research subjects as the European Commission has recently requested (Nature Editorial, 2020), but also regarding the group conducting the research, makes for better science. Following the argument, that approaching the research field and defining the research questions is at least partly dependent upon our experiences in and our views on society leads us to assume that future research could benefit from diversly composed research groups and particularly the inclusion and promotion of women in this research.

## Data Availability Statement

The original contributions presented are included in the article, and further inquiries can be directed to the corresponding author.

## Author Contributions

KL and TN interpreted the literature and drafted the manuscript. All authors approved the final version of the manuscript and agreed to be accountable for the content of the work.

## Funding

The publication of this article was funded by Humboldt-Universität zu Berlin. The funder had no role in study design, data collection, analysis, decision to publish, or manuscript preparation.

## Conflict of Interest

The authors declare that the research was conducted in the absence of any commercial or financial relationships that could be construed as a potential conflict of interest.

## Publisher’s Note

All claims expressed in this article are solely those of the authors and do not necessarily represent those of their affiliated organizations, or those of the publisher, the editors and the reviewers. Any product that may be evaluated in this article, or claim that may be made by its manufacturer, is not guaranteed or endorsed by the publisher.
